# Supplementary figures and images for: The disability-adjusted life years (DALYs), prevalence and incidence of scabies, 1990–2021: A systematic analysis from the Global Burden of Disease Study 2021
Source: PLoS Negl Trop Dis. 2024 Dec 26;18(12):e0012775. doi: 10.1371/journal.pntd.0012775 (PMC11709319; doi:10.1371/journal.pntd.0012775)

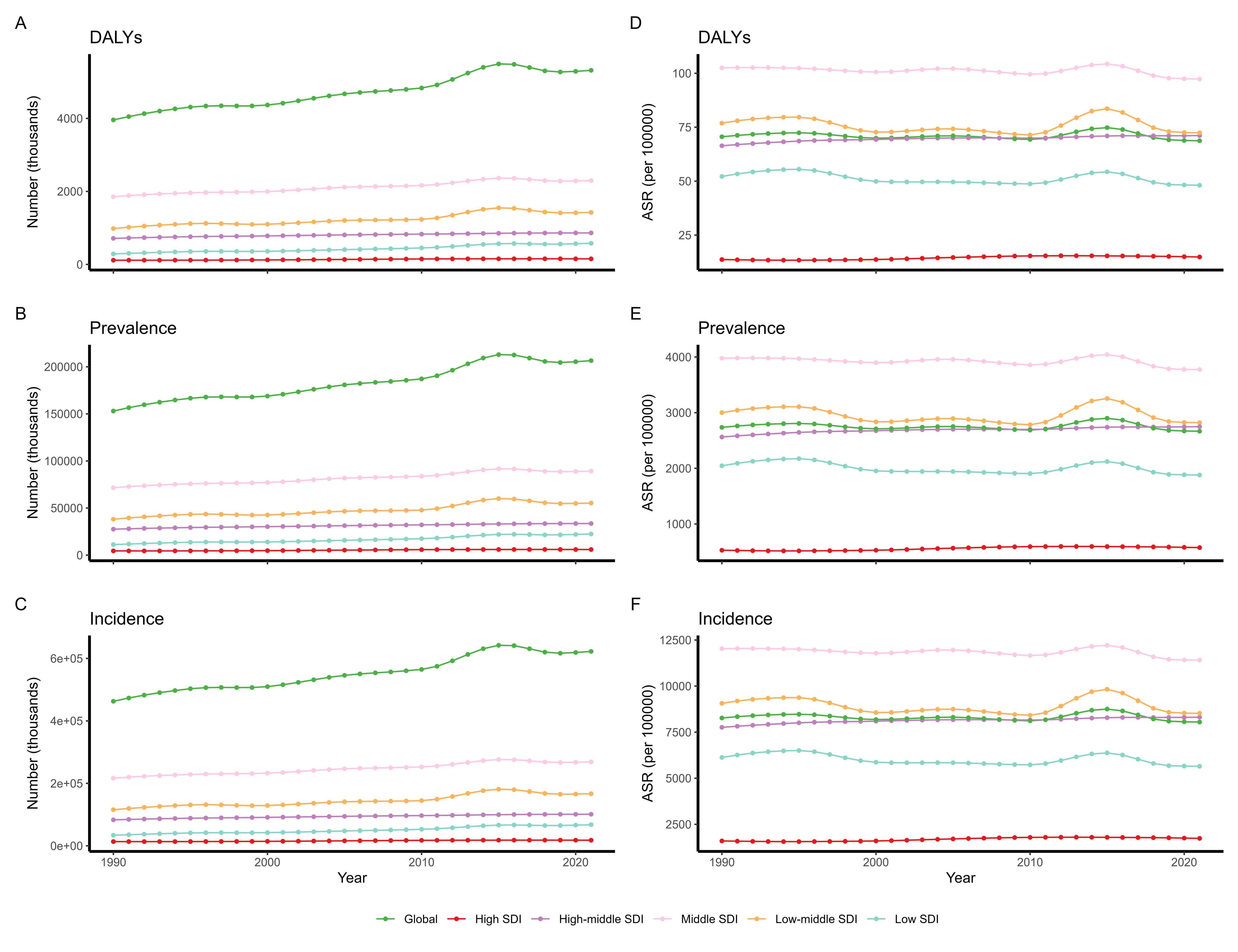

Supplement: S1 Fig — Scabies DALYs (A, D), prevalence (B, E), and incidence (C, F) each year from 1990 to 2021. (TIF) [file pntd.0012775.s001.tif]

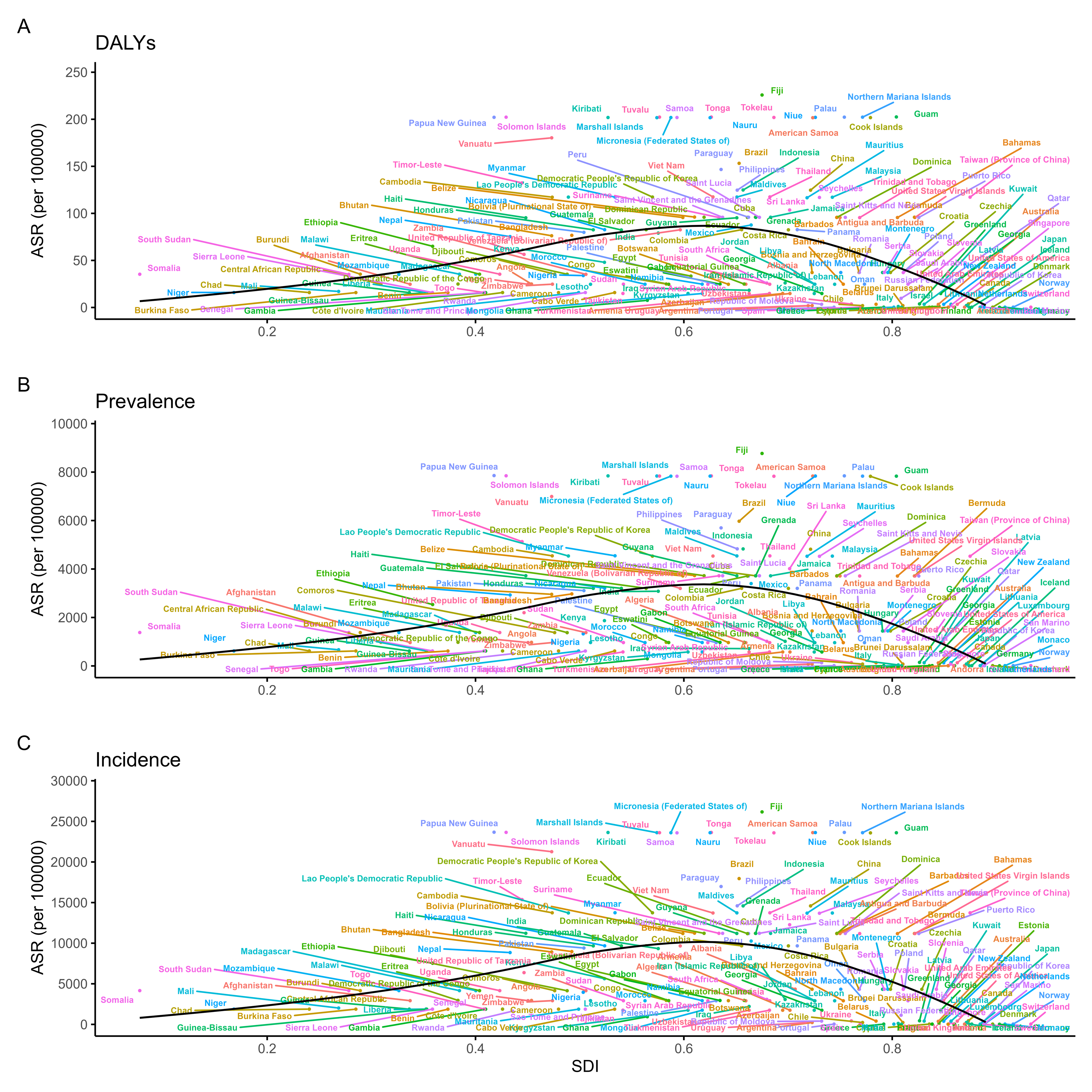

Supplement: S2 Fig — Scabies DALYs (A), prevalence (B), and incidence (C) in ASR across 204 countries and territories by SDI in both sexes, 2021. (TIF) [file pntd.0012775.s002.tif]

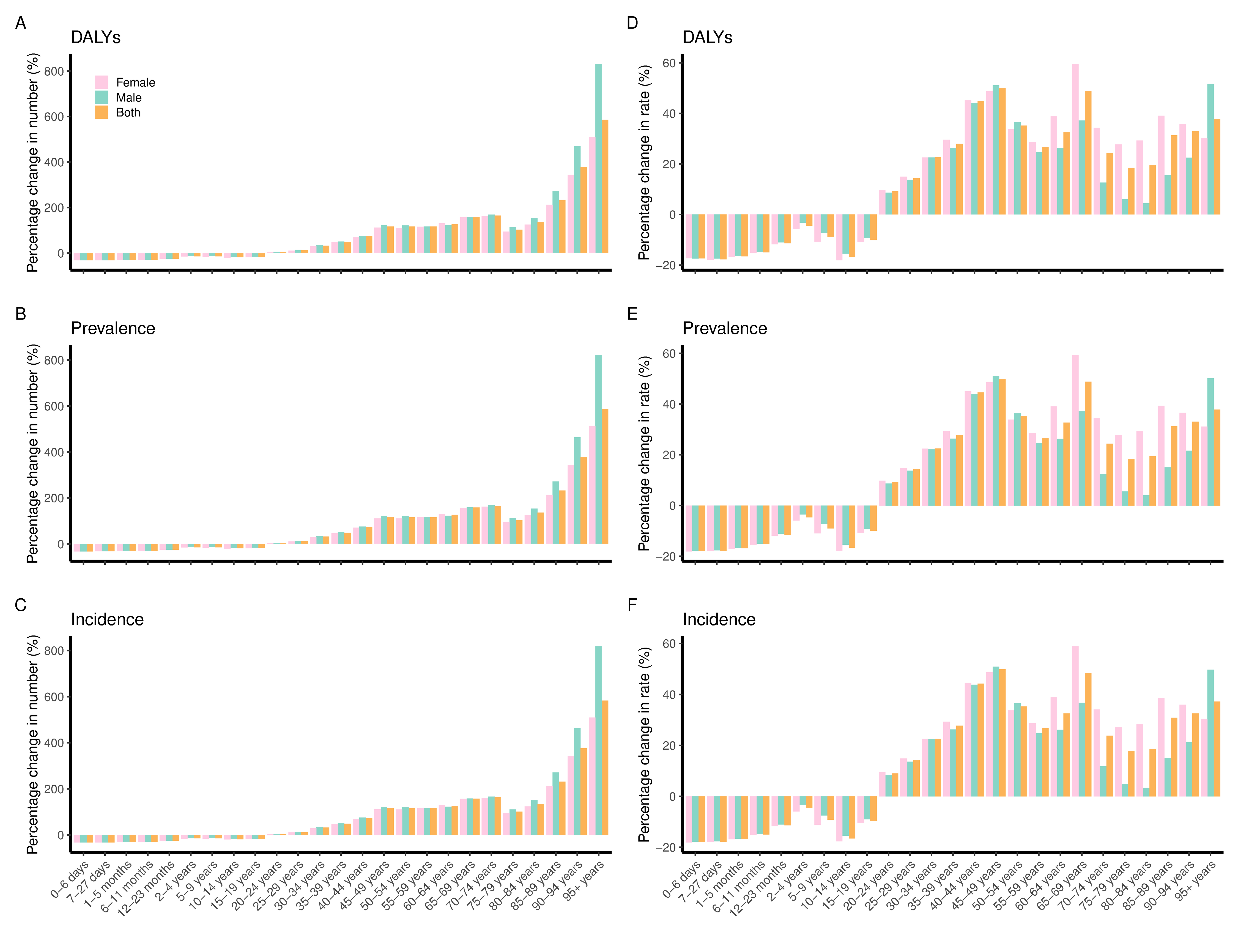

Supplement: S3 Fig — Percentage changes in scabies DALYs (A, D), prevalence (B, E), and incidence (C, F) in high SDI regions from 1990 to 2021. (TIF) [file pntd.0012775.s003.tif]

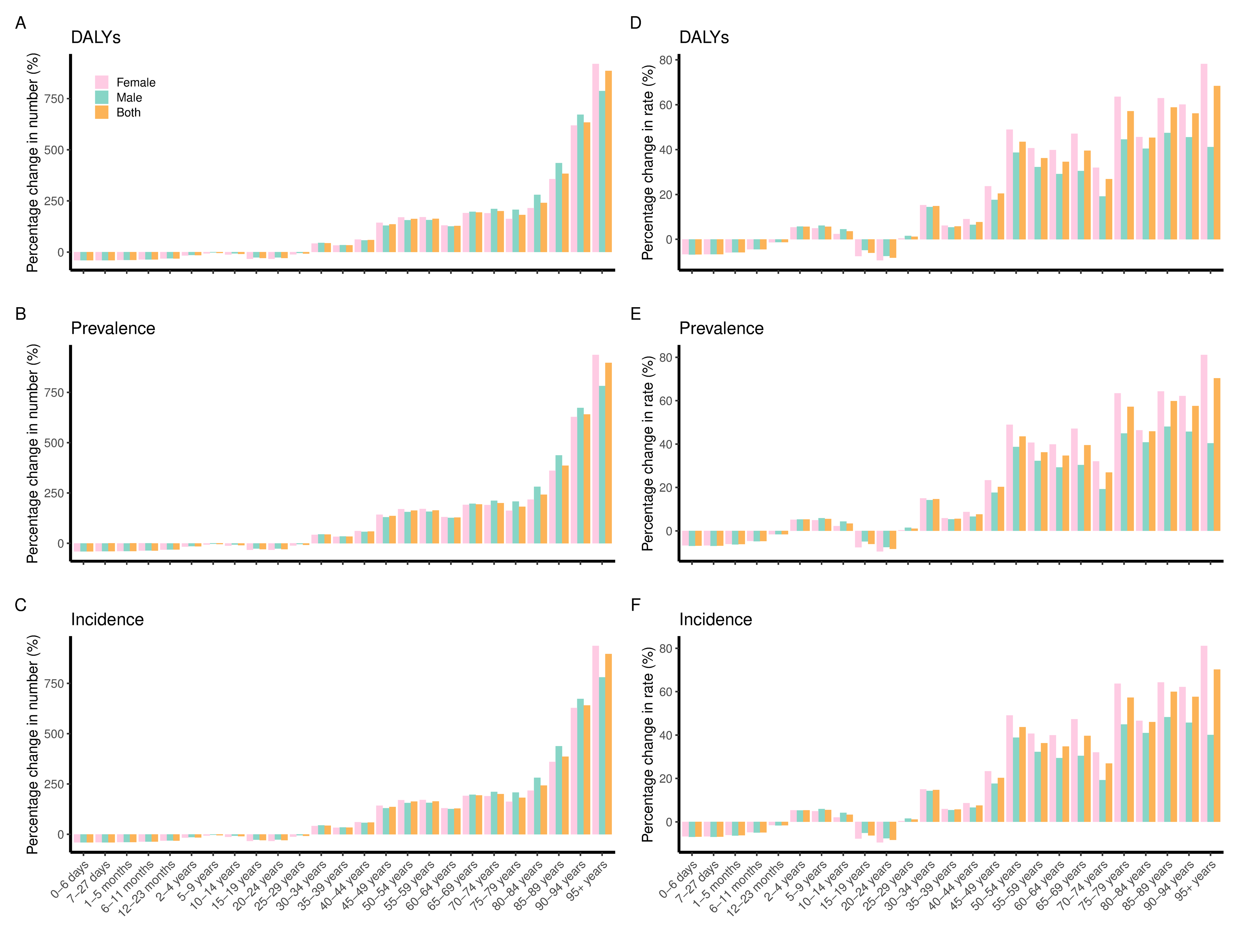

Supplement: S4 Fig — Percentage changes in scabies DALYs (A, D), prevalence (B, E), and incidence (C, F) in high-middle SDI regions from 1990 to 2021. (TIF) [file pntd.0012775.s004.tif]

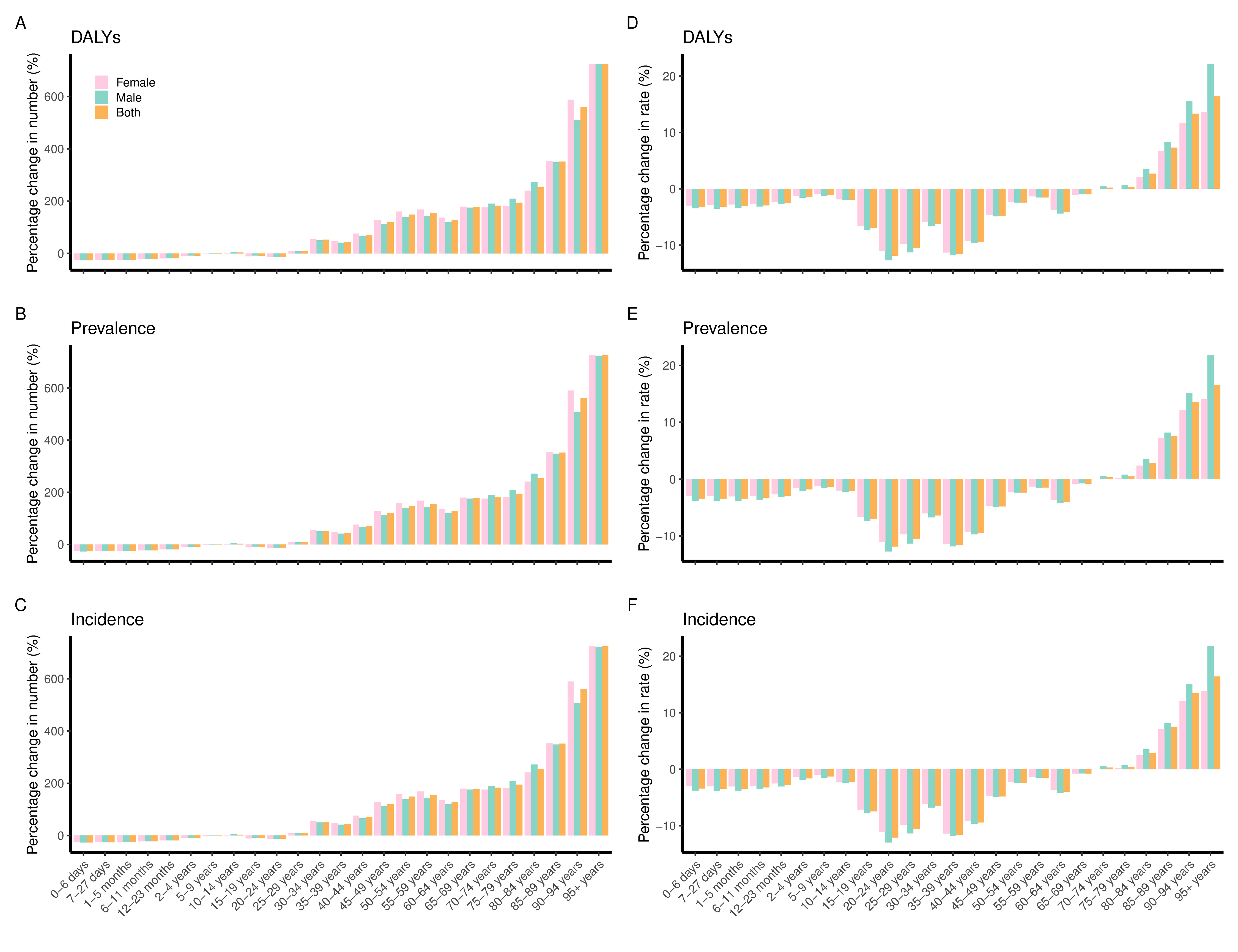

Supplement: S5 Fig — Percentage changes in scabies DALYs (A, D), prevalence (B, E), and incidence (C, F) in middle SDI regions from 1990 to 2021. (TIF) [file pntd.0012775.s005.tif]

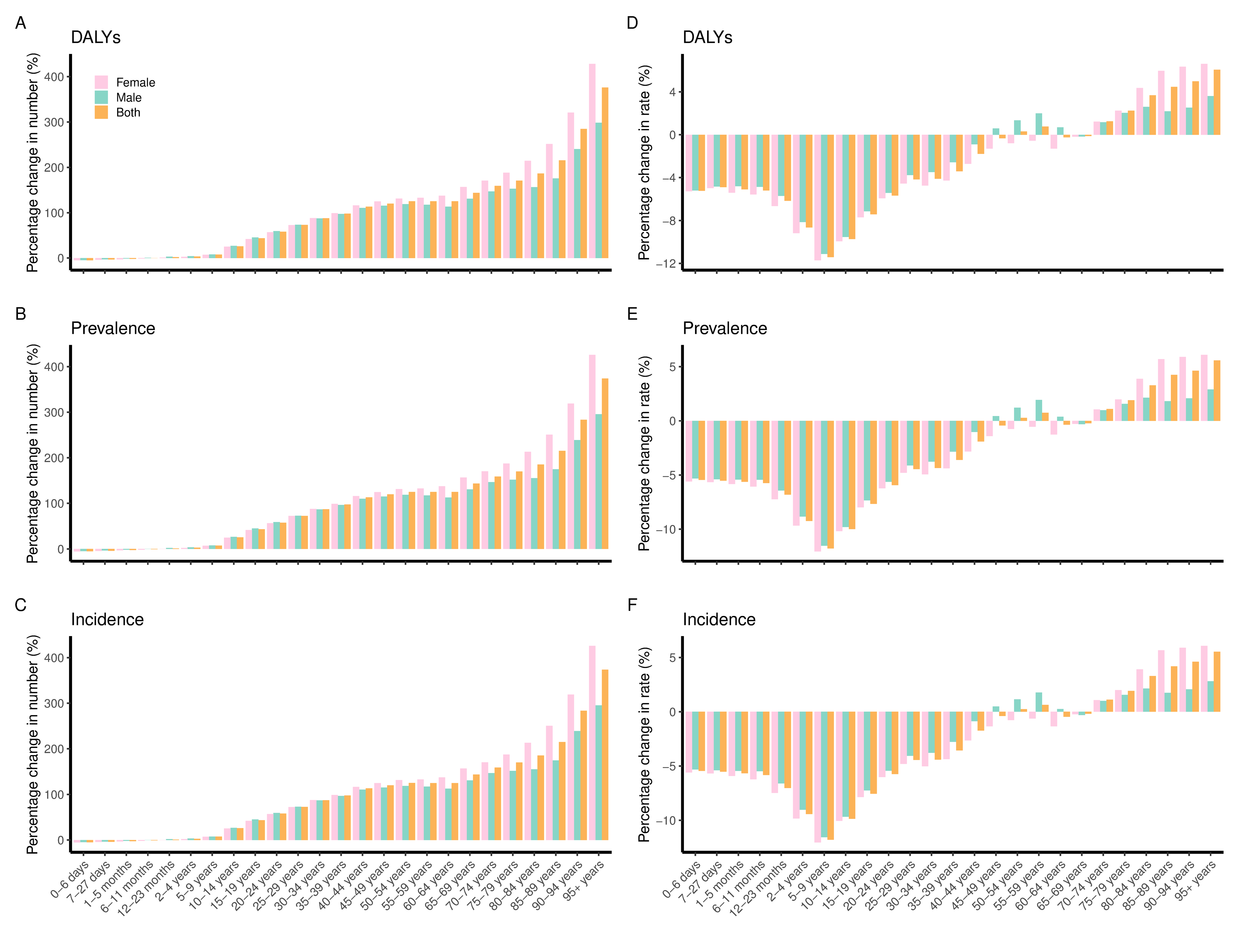

Supplement: S6 Fig — Percentage changes in scabies DALYs (A, D), prevalence (B, E), and incidence (C, F) in low-middle SDI regions from 1990 to 2021. (TIF) [file pntd.0012775.s006.tif]

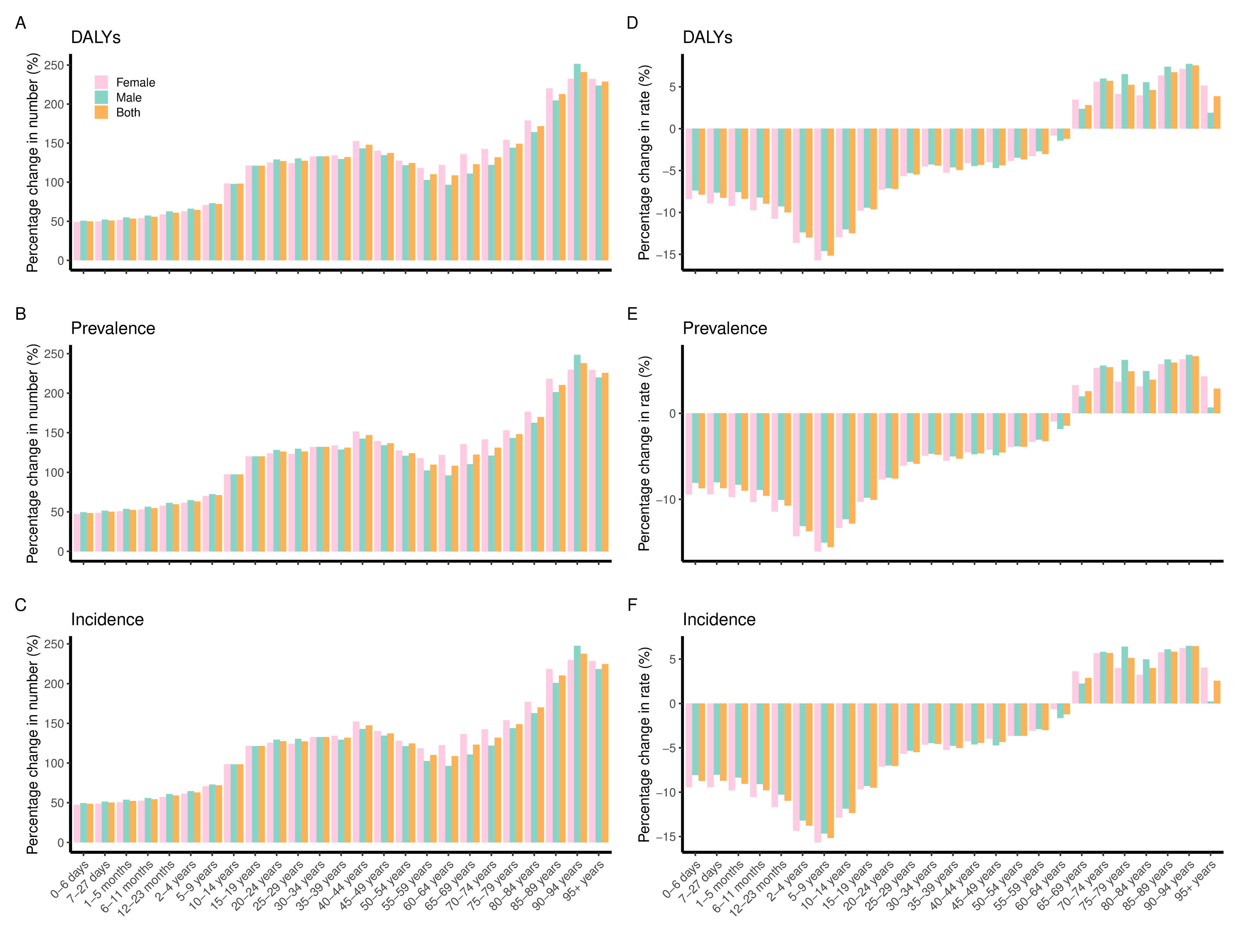

Supplement: S7 Fig — Percentage changes in scabies DALYs (A, D), prevalence (B, E), and incidence (C, F) in low SDI regions from 1990 to 2021. (TIF) [file pntd.0012775.s007.tif]
