# Supplementary material for: The disability-adjusted life years (DALYs), prevalence and incidence of scabies, 1990–2021: A systematic analysis from the Global Burden of Disease Study 2021
Source: PLoS Negl Trop Dis. 2024 Dec 26;18(12):e0012775. doi: 10.1371/journal.pntd.0012775 (PMC11709319; doi:10.1371/journal.pntd.0012775)
Supplement: Attachment — Submitted filename: Letter_Responses to reviewers.docx [file pntd.0012775.s008.docx]

Dear reviewers,

Thank you for your valuable insights regarding our article titled “The disability-adjusted life years (DALYs), prevalence and incidence of scabies, 1990–2021: a systematic analysis from the Global Burden of Disease Study 2021.” We sincerely appreciate your comprehensive review and have carefully addressed each of your points in detail below:

Reviewer #1

Comment 1: The study reported the disease burden only at the beginning and end of the time period without addressing the trends in between. Please include an analysis of the trends over time using the Estimated Annual Percentage Change (EAPC).

Comment 2: Instead of a simple hierarchical analysis, please add the correlation between disease burden and Sociodemographic Index (SDI) at the national level.

Response: For comment 1, we have added an analysis of the trends over time using the Estimated Annual Percentage Change (EAPC) in the revised manuscript. EAPC values are now included in the tables, with corresponding analyses and an introduction in the methods section.

For comment 2, we have added a supplementary figure (S2_Fig) that describes the correlation between disease burden and SDI at the national level.

Reviewer #2

Comment 1: It would be good to clearly articulate what variables from the GBD databased were used for this analysis.

Comment 2: The methods section seems to lack an explanation how DALYs were calculated.

Response: For comment 1, we have clarified the variables used in our analysis in the methods section. “In our analysis, DALYs, prevalence, and incidence attributed to scabies were examined, utilizing both number and rate metrics.” “In our study, burden of scabies was examined across males, females, and both genders combined. All 25 age groups were analyzed. Geographically, the analysis included the globe, five SDI regions, 21 regions, and 204 countries and territories. Temporally, the burden of scabies in 2021 and its changes from 1990 to 2021 were assessed…”

For comment 2, we have provided a more detailed explanation of how DALYs were calculated in the revised manuscript. “...YLDs quantify non-fatal health loss, years of life lost (YLLs) quantify fatal health loss. DALYs are calculated by summing YLDs and YLLs, quantifying both years lost to premature mortality and years lived with disability, enabling comparisons across diseases. For scabies, DALYs were calculated assuming YLLs were zero, making YLDs equivalent to DALYs...”

Reviewer #3

Comment 1: I find it a bit confusing to talk about ‘prevalent cases’ rather than just ‘prevalence’ (same for incidence) and suggest to change wording unless the authors feel strongly about it. Similarly referring to a ‘low burden’ as ‘light burden’ seems odd. Consider changing.

Comment 2: I am wondering if it would be possible to add a temporal analysis based on existing control strategies in the different countries.

Response: For Comment 1, we apologize for any confusion in our word expression. We have revised the wording to use ‘prevalence’ and ‘incidence’ instead of ‘prevalent cases’ and ‘incident cases’ to enhance clarity. Additionally, we have changed ‘light burden’ to ‘low burden’ to ensure clarity and precision in our terminology.

For comment 2, we have added the Fig3, which illustrates the temporal trends in different countries using the Estimated Annual Percentage Change (EAPC).

Reviewer #4

I appreciate the opportunity to critically review the manuscript titled “The disability-adjusted life years (DALYs), prevalence and incidence of scabies, 1990–2021: a systematic analysis from the Global Burden of Disease Study 2021” in which, as the title indicates, the researchers analyzed the most recent GBD data related to scabies. The manuscript is well-written and adheres closely to the methodology of a systematic analysis of GBD data. I present the authors with a series of comments, all of which are minor.

Comment 1: I agree that the GBD methodology, particularly for 2021, has been extensively described in previous publications. However, it is always worthwhile to include a couple of general lines to briefly describe the estimation of the metrics of interest.

Comment 2: What is the disability weight for scabies? If it is low, what are the implications for the obtained metrics? I suggest briefly citing this information.

Comment 3: Lines 352-353 briefly discuss the factors that may be driving the increase in scabies burden in high and upper-middle SDI countries. This is a very important finding that I believe warrants further development. For example, what role do obesity and chronic-degenerative diseases play? What about opportunities for earlier diagnosis in these regions?

Response: For comment 1, we have added some description for DALYs in methods section. “YLDs quantify non-fatal health loss, years of life lost (YLLs) quantify fatal health loss. DALYs are calculated by summing YLDs and YLLs, quantifying both years lost to premature mortality and years lived with disability, enabling comparisons across diseases. For scabies, DALYs were calculated assuming YLLs were zero, making YLDs equivalent to DALYs.”

For comment 2, We have cited this information in our revised manuscript. “Scabies was categorized within the GBD 2021 cause group of skin and subcutaneous conditions and was assigned the disability weight for disfigurement level 1 with itch/pain. The lay description for this level states, “The individual has a slight, visible physical deformity that is sometimes sore or itchy. Others notice the deformity, which causes some worry and discomfort.” Disability weights, ranging from 0 (no health loss) to 1 (equivalent to death), represent health loss from a sequela. The disability weight assigned to scabies was 0.027, which was used to calculate the Years Lived with Disability (YLDs).”

For comment 3, we agree that this is an import finding that warrants further exploration. After carefully reviewing the relevant literature, we have expanded the discussion in the revised manuscript. “In older populations, obesity may exacerbate infections, while dementia or physical limitations caused by chronic-degenerative diseases may delay early diagnosis [18]. This issue warrants further investigation in future research.”

Reviewer #5

Comment 1: Can the authors make clearer what the added benefit of their analysis to the already available results in the GBD results tool is?

Comment 2: In the limitation section the authors state that the countries like Germany don’t report scabies data, but in the results section data for Germany is presented. Please clarify.

Response: For comment 1, firstly, we enhanced the data visualization from multiple perspectives, including calculation of Estimated Annual Percentage Change (EAPC), improving readability and allowing readers to more easily access the information they need. This approach facilitates the broader dissemination and understanding of scabies relevant knowledge through our paper. Secondly, we conducted a systematic and comprehensive analysis of scabies, moving beyond a simple presentation of data to explore the issues it reveals and offering our insights. Finally, we drew specific conclusions that may serve as valuable references for researchers in the field.

For comment 2, there is an explanation in the second paragraph of discussion section. “As scabies was not reportable in Germany, estimates of cases were derived from self-reported scabies, records of drug purchases, statistics from health insurance companies, etc., suggesting that actual figures may be higher.”
