# Supplementary material for: The disability-adjusted life years (DALYs), prevalence and incidence of scabies, 1990–2021: A systematic analysis from the Global Burden of Disease Study 2021
Source: PLoS Negl Trop Dis. 2024 Dec 26;18(12):e0012775. doi: 10.1371/journal.pntd.0012775 (PMC11709319; doi:10.1371/journal.pntd.0012775)
Supplement: Attachment — Submitted filename: Letter_Responses to reviewers.docx [file pntd.0012775.s009.docx]

Dear Editor and Reviewers,

We sincerely appreciate the detailed feedback provided during this second round of revisions regarding our article titled “The disability-adjusted life years (DALYs), prevalence and incidence of scabies, 1990–2021: a systematic analysis from the Global Burden of Disease Study 2021.”

Below, we address each point raised by the reviewers and editorial team:

Reviewer #4

Page 5, line 61. The sentence ends with "et al", but it does not make sense. I think that these words were misplaced and that they can be deleted to end the sentence with "unclear water sources".

Response: We have removed "et al" from the sentence, and it now appropriately ends with "unclear water sources."

Reviewer #5

Many thanks for the opportunity to review this well-thought out analysis. I suggest that the discussion section could be strengthened by discussing the limitations of the data in not being able to account for underdiagnosis of scabies, especially in resource limited settings, and the lack if ability to measure the impact of secondary infections and their sequelae, such as group A strep. This paper neatly discusses these limitations of the epidemiologic data for scabies: Cox V, Fuller LC, Engelman D, Steer A, Hay RJ. Estimating the global burden of scabies: what else do we need? Br J Dermatol. 2021 Feb;184(2):237-242. doi: 10.1111/bjd.19170. Epub 2020 Jul 9. PMID: 32358799.

Response: We fully agree with your observation and have expanded the discussion section accordingly. We also appreciate your reference to the paper 'Estimating the global burden of scabies: what else do we need?' by Cox et al. (2021), which we have now cited in the relevant section to further strengthen our analysis.

“In resource-limited settings, many affected individuals do not seek treatment due to financial constraints and perceptions of care inefficacy. Consequently, reliance on routine clinical data often results in an underestimation of the disease burden. It is likely that the true global prevalence of scabies is considerably higher than current estimates [30] ...... The GBD methodology primarily focuses on the direct effects of scabies, such as itching and skin disfigurement, while failing to account for systemic sequelae resulting from secondary infections caused by Group A Streptococcus, including impetigo, glomerulonephritis, and rheumatic fever, which are frequently associated with scabies [30]. Furthermore, it does not capture mortality associated with crusted scabies [32-34], thus potentially underestimating the overall disease burden.

Reviewer #6

Introduction paragraph: The first paragraph gives a good description of scabies, though the paragraph is somewhat disjointed and I would encourage the authors to reorganize the information here. For instance, lines 85-86 seem out of place (though important to state).

Line 80: I don’t feel the authors need to include species specific bacteria here.

Line 84: is ‘resource-poor’ the preferred term?

Line 95-96: be more specific here; why do the authors feel the efficacy of the various treatment options warrants further investigation.

Response: We have revised the manuscript in response to your constructive feedback. Specifically:

In lines 85-86, we have removed the statement "In 2017, the World Health Organization (WHO) officially classified scabies as a neglected tropical disease (NTD), urging more attention from society [3]".

In line 80, we have removed the reference to Staphylococcus aureus and Streptococcus pyogenes as it was not necessary to mention species-specific bacteria in this context.

In line 84, we have replaced the term "resource-poor" with "resource-limited," which we believe is a more widely accepted and precise term in the context of global health and healthcare access.

In lines 95-96, we have expanded on the explanation regarding the need for further investigation into treatment efficacy. The sentence now reads: "While scabies remains treatable with medications such as ivermectin, permethrin, benzyl benzoate, and crotamiton, reports of reduced in vitro susceptibility and clinical resistance to the primary scabicides, permethrin and ivermectin, have emerged. These observations, coupled with concerns about treatment efficacy in recent years, underscore the need for further investigation into the underlying causes of potential resistance and treatment failure [5]."

Results section: The authors present a detailed report of scabies DALYs over the past 3 decades using GBD data. Given the change in population over this period of time, it may be helpful to present incidence as cases per 100,000 people in the regional, national sections. Overall it is a detailed description of the global burden of scabies and the figures provide good representation of the data.

Line 200: I would suggest using another term than “most severe”

Response: Regarding the incidence rate, we have already included this metric in the relevant sections to ensure a standardized comparison across regions and countries. For instance, Table 3 features a column titled “ASR (age-standardized rate [per 100,000]) in 2021 (95% UI),” which provides detailed incidence rates. Additionally, we describe these metrics in the text, such as: “The ASRs of DALYs, prevalence, and incidence in middle SDI regions were 97.3 (54.4–160.0), 3774.7 (3361.3–4223.8), and 11,409.1 (10,174.5–12,796.2), respectively.” We hope this clarifies how incidence rates are presented throughout the manuscript.

Additionally, we have revised the sentence to replace "most severe" with "highest" to provide a clearer and more appropriate description of the burden. The updated sentence now reads: "In 2021, the burden of scabies was highest in middle SDI regions and lowest in high SDI regions."

Conclusions section: Lines 373-375: Authors state that Central America has had a higher burden of scabies and suggest it may be related to a decline in socioeconomic conditions. Though the paragraph starts with a discussion of scabies incidence on a national level. Can the authors report more on the country specific data in Central America and if this correlates with changes in socioeconomic status, recognizing that not all countries in Central America are suffering from suboptimal socioeconomic conditions.

Lines 378-382: authors make statements regarding the possibility of a decline in the efficacy of first line scabies treatments; this should be developed further in the discussion. In addition, I would suggest expanding the discussion on how scabies incidence may be impacted by migration.

403-404: authors suggest that post-menopausal women may have an increased risk of scabies due to changes in the skin; if the authors are going to include this here they need to provide more substantial evidence to support this claim.

411-425: Authors discuss the limitations of this study, and I would suggest further discussion of the impact of under-reporting of scabies, particularly in LMICs.

Response: For comment 1 (Lines 373-375). We have provided detailed country-specific data on DALYs, prevalence, and incidence in Figures 1-3. Additionally, the relationship between sociodemographic index (SDI) and the age-standardized rates (ASRs) of DALYs, prevalence, and incidence at the national level is illustrated in S2_Fig. As described in the manuscript: "Regarding the relationship between SDI and the ASRs of DALYs, prevalence, and incidence at the national level, it was observed that as SDI increased, these ASRs followed a pattern of initially rising and then declining, with the highest ASRs primarily concentrated in countries with medium to upper-middle SDI levels. This pattern was consistent with the trends previously described at the SDI regional level." We fully agree with your observation that "not all countries in Central America are suffering from suboptimal socioeconomic conditions." To reflect this, we have adopted a stratified approach in the manuscript, presenting the disease burden at global, regional, and national levels. This structure allows for a clearer depiction of the overall burden at regional level while highlighting the specific burden in individual countries.

For comment 2 (Lines 378-382). About ‘the possibility of a decline in the efficacy of first line scabies treatments’, we have expanded the discussion as follows: “Predictors of treatment failure include the immune status of the host, the choice of treatment, exposure to future transmission events, and potential drug resistance. Although no conclusive evidence currently exists to confirm scabies resistance to frontline scabicides, such as permethrin and ivermectin, skepticism about their efficacy has grown in recent years, highlighting the need for further studies to investigate the underlying causes of treatment failure [5].” Additionally, in the conclusion section, we have mentioned that combination therapy and the development of new scabies treatments may be helpful in controlling scabies. Regarding ‘how scabies incidence may be impacted by migration’, we have expanded the discussion as follows: “Furthermore, travel and refugee movements contribute to the spread of scabies in European regions, driven by population influxes from areas with high scabies prevalence and overcrowded living conditions that promote direct and prolonged skin-to-skin contact [3].”

For comment 3 (Lines 403-404). We understand that the hypothesis regarding an increased risk of scabies in post-menopausal women due to skin changes requires more substantial evidence. Given the limitations of the current literature, we have revised the relevant section to present this idea more cautiously and have explicitly stated that further research is needed to validate this hypothesis. “Female skin tends to be thinner compared to males, particularly post-menopause, which might influence skin resistance [26]. However, further research is needed to confirm these observations and elucidate the underlying mechanisms.”

For comment 4 (Lines 411-425). Thank you for highlighting the importance of under-reporting, particularly in resource-limited settings. We have expanded the discussion：Specifically, we highlighted financial barriers and perceptions of care inefficacy, which deter affected individuals from seeking treatment, leading to an underestimation of the disease burden. These challenges emphasize the need for increased awareness and improved access to prevention and treatment resources in affected regions, which could help mitigate the impact of under-reporting.

We hope these explanations sufficiently addresses your concerns.

Reviewer #7

No major recommendations from my end, only would note that et al. appears twice in the paper with no names preceding - lines 61 and 410.

Response: We have carefully reviewed the manuscript and deleted the "et al." appeared in lines 61 and 410.

Congratulations to the authors on what seems to be a very comprehensive analysis. Although the public health relevance is definitely there, I found little novelty with the conclusions of the research to the field of scabies epidemiology research. Not to say this research is not worthy of publishing, I do wonder on the scope of its contribution to the sector.

Response: Thank you for your comment and for recognizing the public health relevance of our study. As stated in the manuscript, our analysis provides a comprehensive overview of the global burden of scabies and its recent trends. We believe this information may serve as a valuable reference for developing region-specific scabies prevention and control measures. While we acknowledge the limitations in novelty, we hope the detailed data and insights presented will contribute to ongoing efforts in scabies research and public health planning.

We are grateful for the opportunity to improve the manuscript further. We hope it now meets the journal's standards for publication.

Thank you again for your thoughtful review and guidance. We look forward to your decision.
